# Effects of Foliar Treatment with a *Trichoderma* Plant Biostimulant Consortium on *Passiflora caerulea* L. Yield and Quality

**DOI:** 10.3390/microorganisms8010123

**Published:** 2020-01-16

**Authors:** Tatiana Eugenia Şesan, Anca Olguța Oancea, Laura Mihaela Ştefan, Vasile Sorin Mănoiu, Marius Ghiurea, Iuliana Răut, Diana Constantinescu-Aruxandei, Agnes Toma, Simona Savin, Adriana Florina Bira, Cristian Mihai Pomohaci, Florin Oancea

**Affiliations:** 1 Department of Botany and Microbiology, Faculty of Biology, University of Bucharest, Aleea Portocalilor nr. 1-3, sector 6, 060101 Bucharest, Romania; tatianasesan@yahoo.com; 2 Departments of Biotechnology and Bioresources, National Research & Development Institute for Chemistry and Petrochemistry—ICECHIM, Splaiul Independenței nr. 202, sector 6, 060021 Bucharest, Romania; ghiurea@gmail.com (M.G.); iulia_rt@yahoo.com (I.R.); diana.c.aruxandei@gmail.com (D.C.-A.); 3 Department of Cellular and Molecular Biology, National Research & Development Institute for Biological Sciences, Splaiul Independenței 296, sector 6, 060031 Bucharest, Romaniaspagiricus@yahoo.com (V.S.M.); agnes12ro@yahoo.com.au (A.T.); simonatulea@yahoo.com (S.S.); 4 Department of Research & Development, Hofigal SA, Intrarea Serelor, Nr. 2, Sector 4, 042124 Bucharest, Romania; adrianaflorinabira@yahoo.com; 5 Department of Mathematics, Physics and Land Measurements, Faculty of Land Reclamation and Environmental Engineering, University of Agronomical Sciences and Veterinary Medicine, Bulevardul Mărăști 59, sector 1, 011464 Bucharest, Romania; informatico@gmail.com; 6 Biotechnologies Department, Faculty of Biotechnologies, University of Agronomical Sciences and Veterinary Medicine, Bulevardul Mărăști 59, sector 1, 011464 Bucharest, Romania

**Keywords:** phyllosphere colonization, spores concentration, leaf area, leaf ultrastructure, polyphenols and flavonoids, antioxidant activity, stomatal conductance, chlorophyll fluorescence, cells culture biocompatibility, crop yield and quality

## Abstract

The influence of spore concentration on the ability of a *Trichoderma* consortium to colonize the *Passiflora caerulea* phyllosphere was evaluated by determining the effects of foliar treatments with two spore concentrations, in two repeated treatments, on the morphological, physiological, and ultrastructural characteristics, and on the yield and quality of *P. caerulea*. The studied crop quality features were related to its nutraceutical use: the accumulation of polyphenols and flavonoids, antioxidant activity, and effects on mouse fibroblast L929 cells. The *Trichoderma* consortium consisted of two strains, *T. asperellum* T36b and *T. harzianum* Td50b, and the concentrations used were 10^6^ colony forming units (cfu)/mL and 10^8^ cfu/mL. As a reference treatment, a commercial product that was based on herbs and algal extracts was used. As compared to the negative control, the treatment with the *Trichoderma* consortium at 10^8^ cfu/mL concentration determines the accumulation of higher level of polyphenols and flavonoids and increased antioxidant activity. This enhancement of *P. caerulea* quality characteristics after treatment with the higher concentration of *Trichoderma* consortium was associated with larger leaves, increased number and size of chloroplasts, improved plant physiology characteristics, and an increased yield. The treatment with high concentration of *Trichoderma* consortium spores promotes phyllosphere colonization and benefits both crop yield and quality.

## 1. Introduction

Plant-beneficial fungi from the *Hypocrea*/*Trichoderma* genera are among the most widespread microorganisms used in agriculture [1]. Selected and highly active strains are applied as seed [2], soil [3], and foliar treatments [4], and they are largely used as bio/myco-fungicides [5,6], plant biostimulants [7], and biofertilizers [5,8]. The multifaceted actions of plant-beneficial *Trichoderma* strains are determined by several mechanisms, which are mainly related to antagonism against plant pathogens [9] or activation of plant defence [10]. Such multifunctional strains produce hydrolases with lytic effects on plant pathogens [11] and/or that release specific oligosaccharide elicitors (damage-associated molecular patterns; DAMPs) from plant cell walls [12]. Several strains produce hydrolases together with proteins from the cerato-platanin family, weakening fungal cell walls and acting as microbial elicitors (pathogen or microbial-associated molecular patterns; PAMP, or, respectively, MAMPs) [13,14], as well as secondary metabolites, including peptides and volatile compounds, acting against fungal pathogens [11], activating plant defence response to stress [15], and/or improving plant root system morphology and physiology [16,17,18].

The activation of plant defence following *Trichoderma* treatments not only reduces plant diseases. In tomatoes, the defence barriers against both chewing herbivore insects (*Spodoptera littoralis*) and piercing–sucking aphids (*Macrosiphum euphorbiae*) were activated after *Trichoderma atroviride* P1 colonization [19]. *Trichoderma* application increased the plant tolerance to abiotic stress, such as drought [20], salinity stress [21], low temperatures [22], and recalcitrant pollutants [23]. The application of *Trichoderma* strains increases plant photosynthetic ability, up-regulating both light harvesting components and Calvin cycle (dark reaction) components [24]. *Trichoderma* and its secondary metabolites also enhance the uptake and use efficiency of macronutrients, such as nitrogen [3,16] or phosphorus [25,26], and oligo/micro-nutrients, such as iron [8,27,28] or zinc [29].

In addition to higher yield, the treatment with plant-beneficial *Trichoderma* strains also contributes to enhanced nutritional quality [30]. This is because the activation of the plant defence response by *Trichoderma* strains is associated with higher accumulation of plant secondary metabolites, which are involved in plant defence against biotic and abiotic stress [31]. Such secondary metabolites are considered to be important phytonutrients for humans, with a significant function in maintaining human health [32]. Illustrative examples are artemisin, which accumulates in larger quantities in *Artemisia annua* following *Trichoderma* treatment [33], and bioactive compounds, including lycopene, which is higher in tomatoe fruits that are produced from *Trichoderma*-treated plants [34]. Treatment with *Trichoderma* strains, especially with those selected for their plant defence-triggering effects, was demonstrated to increase the polyphenolic content of various plants—grape [35], artichoke [36], and tomatoes [20]. Flavonoids and polyphenols were shown to increase the edible parts of onions [37] and cucumbers [38] after treatment with *Trichoderma* strains.

Plants from the *Passiflora* genus, which are commonly named passion fruits/passion flowers, are well-known for their effects on human health and wellbeing. The edible parts are considered to be nutraceutical/functional food [39]. The aerial parts of *Passiflora* plants are used as infusions, extracts, or tinctures due to their various effects: anti-depressive/anti-anxiolytic [40,41]; sedative/anti-sleep disorders [42]; anti-spasmolytic [43]; anti-asthma/anti-respiratory disorders [44]; anti-diabetic/hypolipidemic [45]; anti-hypertensive [46]; and, anti-addictive [47,48]. These effects have been related to the bioactive polyphenols and flavonoids [49,50] and their antioxidant [51,52,53], antimicrobial [52], prebiotic [51], and anti-inflammatory and analgesic [54,55,56] activities. To date, strains from *Trichoderma* genera were applied to *Passiflora* plants as bio/myco-fungicides for the treatment of fungal diseases [57,58] or as biofertilizer to promote root growth and root function related to mineral nutrient uptake [59]. There are no studies regarding the influence of foliar treatments with *Trichoderma* strains on the accumulation of bioactive compounds in the aerial parts of the passion plants (largely used for nutraceutical purposes).

In this study, we determined the effects of foliar treatments with a *Trichoderma* consortium on the yield and quality of blue passion flowers. Such effects, which are specific for microbial plant biostimulants, are determined by the ability of the applied beneficial microorganisms to colonize the specific plant microbiocenosis, e.g., in the rhizosphere or phyllosphere, in competition with already existing microbes [60]. One strategy to promote inoculated *Trichoderma* capacity to colonize the plant microbiocenosis is that of repeated applications of the active strains, e.g., the highly effective *T. harzianum* strain T22 (ATCC^®^ 20847™) was applied 10 times every two weeks at a density of 10^8^ spores per liter to *Vitis vinifera* cv. Sangioves to induce the accumulation of polyphenols in grapes [26]. Such strategy, despite its success, is difficult to scale-up due to the costs involved by the application of 10 treatments, even in the case of profitable horticultural crops.

The main aim of our study was to test another strategy, easier to scale-up, for the phyllosphere colonization with the applied beneficial microorganisms—a higher concentration of a *Trichoderma* consortium, containing a larger amount of chlamydospores [61], spores more resistant to adverse conditions in comparison to dried mycelia, and conidiospores, being applied in a lower number of treatments. The hypothesis was that the more resistant spores, from two compatible strains, will last longer in the (rather) hostile (for fungi) leaf habitat, having more chances for successful phyllosphere colonization. We tested two levels of spore concentration, 10^6^ and 10^8^ spore/mL, applied as two treatments, and we determined the effects of the treatments on the yield and quality of *Passiflora caerulea*—polyphenol and flavonoid accumulation and antioxidant activity of leaves. Morpho-physiological determinations (leaf development and ultrastructure, leaf chlorophyll fluorescence, and stomatal conductance) were performed to demonstrate the colonization of the phyllosphere with the *Trichoderma* consortium and substantiate the effects on *P. caerulea* yield and quality.

## 2. Materials and Methods

### 2.1. Biological Material

*Passiflora caerulea* plants (blue passion flower) were grown in the Hofigal experimental field located south of Bucharest (44°25’15” N, 26°1’34” E, altitude 84 m) on a reddish preluvosol. The average values of multi-annual temperature, wind speed, daily sunshine duration, and total precipitation for this experimental site are 11.5 °C, 3.2 m s^−1^, 6.8 h, and 615 mm, respectively. The respective average multi-annual monthly temperature and precipitation are as follows: April—13.8 °C and 78.7 mm; May—16.1 °C and 84.2 mm; June—25.7 °C and 53.6 mm; July—27.5 °C and 42.7 mm; August—25.2 °C and 68.7 mm. During the experimental period, some differences compared to the multi-annual average were recorded. The average monthly temperatures were generally higher (+1.1 °C in April; +0.7 °C in May; +2.3 °C in June, −0.2 °C in July, +0.4 °C in August) and the average monthly precipitation was generally lower (−11.4 mm in April; −12.4 mm in May; –9.6 mm in June; +3.2 mm in July; –4.8 mm in August) than the multi-annual average. The experimental field was irrigated up to 80% field capacity during the whole vegetation period of *Passiflora caerulea* plants. For this study, a consortium formed of two strains from the INCDCP-ICECHIM collection, *T. asperellum* T36 NCAIM F 001434 and *T. harzianum*, Td50b, NCAIM F001412 was used as the microbial plant biostimulant. Both of the strains are multifunctional plant biostimulant strains. Both strains produce bioactive volatile compounds, including 6-pentyl-2H-pyran-2-one-6-PP [62,63]; both are antagonist against major plant pathogens [63,64], protect and stimulate vegetable growth [65], accelerate the degradation of lignocellulose material, and promote development in early stages of the plants that are cultivated in high residue systems, i.e., mulch produced from winter cover crops [66,67]. This highly compatible consortium was grown in a medium promoting chlamydospore accumulation: 34.2 g/L glucose, 0.37 g/L ammonium sulphate, 0.8 g/L yeast extract, 2.7 g/L soymeal, 1.2 g/L K_2_HPO_4_, and 1.7 g/L KH_2_PO_4_ [68] at 25 °C, with light (16 h day/8 h night) for two weeks. The mycelium was homogenized with the culture medium by vigorous shaking, and the resulting suspension was aseptically poured through a 100-mesh cotton tissue. The spores were collected in a sterile bottle and then concentrated by centrifugation [69]. The concentration of the *Trichoderma* consortium propagules was normalized to 10^6^ and 10^8^ cfu/mL by microscopic counting of fungal spores on a hemocytometer according to a standardized protocol prior to the application as treatment of *P. caerulea* plants [70].

### 2.2. Field Experiment

The experiment included three different foliar treatments, which were compared with the control, as follows: C—control (no treatment with plant biostimulants; treated only with water, volume equivalent to 200 L/ha); T_1_—foliar treatment with *Trichoderma* consortium suspension, 10^6^ cfu/mL, with a normalized spraying volume equivalent to 200 L/ha corresponding to a dose of 2 × 10^11^ spores/ha; T_2_—foliar treatment with *Trichoderma* consortium suspension, 10^8^ cfu/mL, spraying volume 200 L/ha, equivalent to a dose of 2 × 10^13^ spores/ha; and, T_3_—foliar treatment with a reference product (Amalgerol, Hechenbichler, Innsbruck, Austria) that contains plant extracts, essential oils, and fatty acids in an oil/water emulsion and extract of the seaweed *Ascophyllum nodosum* applied at 1.2% concentration at a normalized spraying volume of 250 L/ha, corresponding to a dose of 3 L/ha. The reference product was selected because it was demonstrated to significantly enhance the accumulation of polyphenols [71] and contains both elicitors (from herbs and seaweed extract) and volatiles (essential oils). The spore concentrations of the *Trichoderma* consortium were selected in accordance with the demonstrated effects on *P. caerulea* morphology [72]. The experiment was randomized in a Latin square design and each treatment had four repetitions. Each experimental repetition included 50 *Passiflora* plants. The two treatments were applied using an SG20 backpack sprayer (Stihl AG, Waiblingen, Germany) at the beginning of June (leaf development and flower primordium formation) and at the beginning of July (flower development). The applied pressure of the back-up sprayer was set to 275 kPa. A nozzle with a flat jet and low drift (TeeJett^®^ flat-fan TT11002 model, Spraying Systems, Wheaton, IL, US) was used.

### 2.3. Determination of Morphological and Physiological Characteristics of P. caerulea Plants

The *P. caerulea* plants were monitored during the whole experiment. Their morpho-physiological parameters were measured one day before the application of the first treatment, 7 days after the application of the first treatment and at the end of the experiment—at the beginning of August, 60 days after the application of the first treatment. *P. caerulea* leaves were sampled for biochemical analysis of bioactive compounds and for alternative tests on cell cultures on the same dates when the morphological and physiological characteristics were assayed. At the end of the experiment, the yield level was evaluated as the marketable biomass of aerial parts for each plot/repetition (15 plants harvested per repetition; plants were not previously sampled for aerial parts). The marketable biomass of *P. caerulea* represents 50% of the aerial parts of the nutraceutical plant, which can be harvested without compromising any further development of this perennial plant and it is marketable to produce infusions, extracts, or tinctures.

10 healthy leaves per repetition were randomly collected (in total, 40 leaves per experimental treatment) on each sampling date for the estimation of the average leaf area (LA). The leaf area (cm^2^) was evaluated by two different methods, a classic one, using calculation and interpolation on squared paper, and an image analysis method, as already described [73]. Briefly, for the classic method, drawings of scanned fresh leaves on an A4 sheet of paper divided into squares of 5 mm were made. For the determination of the leaf surface by image analysis, the morphometric methods that were based on multiscale Minkowski fractal dimension [74] and Quick Photo Micro 2.3 software (Promicra, Prague, Czech Republic) were used. The classical method for calculation of the leaf area involves the use of the length (L) and width (w) of the leaf, according to the following equation [75]:

LA = L× w × k.(1)

In Equation (1), k is a coefficient specific for each plant species/variety and plant development stage. The length, width, and area of each of the scanned fresh leaves on squared paper were measured. The resulting k coefficient was calculated and statistically analyzed.

The measurement of chlorophyll fluorescence of *Passiflora* plants was undertaken with a PAM fluorometer (Walz PAM 2500, Effertlich, Germany), according to the manufacturer’s instruction. The determinations were done on 10 leaves that were randomly chosen per repetition (selected from representative upper leaves of healthy plants from each repetition). Before starting the measurement, the leaves were pre-darkened for 30 min. by using a brown paper bag, after which saturation light pulses were applied. The maximum photosystem II (PSII) quantum efficiency, i.e., the ratio between F_v_, variable fluorescence, and F_m_, the maximum fluorescent yield in the dark-adapted state, was determined. The stomatal conductance (nmol m^−2^ s^−1^) of the *P. caerulea* leaves was measured with a Delta T AP4 porometer (Delta-T Devices, Burwell, UK) while using the same leaves on which the chlorophyll fluorescence was measured. Each determination was repeated three times.

### 2.4. Ultrastructural and Morphological Analyses

A Philips EM 208S electron microscope (Philips Electron Optics, Amsterdam, Netherland) equipped with a Veleta video camera and imaging software iTEM (Olympus Soft Imaging Solutions, Münster, Germany) was used to investigate the ultrastructure of *P. caerulea* leaves. The samples were submitted to a 2-h plant-specific fixation process while using 1.5% paraformaldehyde, 1 M Na_3_PO_4,_ and 3% glutaraldehyde. The fixation step was followed by rinsing with 0.5 M Na_3_PO_4_ (three times at 4 °C). For the post-fixation step, the sample was transferred to the dark for 1 h in a solution of 1% OsO_4_ and 0.5 M Na_3_PO_4_. After the post-fixation step, the samples were washed three times for 10 min. in water at 4 °C and were then dehydrated by successive washing with ethanol for 10 min. each: 12.5%, 25%, 35%, 50%, 70%, 80%, 90%, 95%, and 100%. To replace the old solution and ethanol completely with an acetone-resin mixture, the dehydrated samples were incubated successively with acetone-resin (1:1) for 1 h, acetone-resin (1:2) for ½ h, resin 100% for 1 h, and then fresh resin 100% overnight. The leaf samples were placed in fresh resin (100%) for 60 h at 50 °C to perform the polymerization step. The resin-embedded samples were cut with an ultramicrotome Leica UC6 (Leica Biosystem, Wetzlar, Germany) with a diamond knife, and the sections were placed on copper grids of 200 mesh that were covered with a formvar pellicle. The samples were stained for 7 min. with 5% uranyl acetate in absolute methanol to increase the contrast. Subsequently, the samples were washed in distilled water and then re-stained for another 7 min., with a solution of lead nitrate 4.4% and 0.2 M trisodium citrate monohydrate in distilled water (with 1% NaOH to clarify the solution). All of the reagents were of electron microscopy grade, supplied by Sigma–Aldrich (Sigma, Merck Group, Darmastad, Germany).

### 2.5. Determination of Chloroplast Number and Their Surface in the Spongy Parenchyma

The ultrathin sections of the leaves from control and plant biostimulant treatments were evaluated with transmission electron microscopy (TEM) to determine the changes in the ultrastructure of the spongy parenchyma and chloroplasts, as well as in the number of chloroplasts and their surface. These parameters were determined according to Zechman [76]. Briefly, 100 cells from the TEM images of spongy parenchyma from each replicate were visually inspected and the number of chloroplasts was determined by counting. The chloroplast surface was determined by image analysis. The perimeter of the chloroplast was manually tracked and the surface (μm^2^) of the structure was determined while using calibrated ImageJ software 1.52P (http://rsbweb.nih.gov/ij/). The examination for chloroplast structure evaluation was undertaken for chloroplasts included into 10 different cells from four different samples per each replicate.

### 2.6. Determination of Total Polyphenols and Flavonoids

The sampled fresh plant material (leaves and sprouts with leaves) was dried at 50 °C for 24 h, until 6–7% final moisture content was achieved, and then ground in a laboratory mill (Retsch SM2000 (Retsch GmbH, Haan, Germany) fitted with a 1 mm sieve. The resulting ground plant material was macerated in ethanol (EtOH) solution in pure water, 70% (*v*/*v*) for 10 days. After 10 days, the macerate was filtered through a vacuum filter, and the resulting filtrate was stored at 4 °C in dark bottles. In the ethanol macerate, the total polyphenols were determined while using the Folin–Ciocâlteu method [77], with some modifications [78]: 750 µL of Folin–Ciocâlteu reagent, 4 mL of 15% Na_2_CO_3_, and distilled water were added to 150 µL of sample to a final volume of 15 mL. The absorbance at λ = 756 nm was measured after 2 h incubation at room temperature. The total phenolic compounds were expressed as gallic acid (GA) equivalents based on a calibration curve that was constructed with known concentrations of gallic acid. The aluminum chloride colorimetric method was used for total flavonoids assay [79]: 0.5 mL of sample was mixed with 1.5 mL ethanol, 0.1 mL of 1 M potassium acetate, 0.1 mL of 10% aluminum chloride, and 2.8 mL of distilled water; the absorbance at λ = 415 nm was measured after 30 min. incubation at room temperature. The flavonoid content was expressed as quercetin (Q) equivalents while using a calibration curve constructed with known concentrations of quercetin. All of the determinations were done in triplicate. All of the reagents used were analytical-grade reagents purchased from Sigma–Aldrich (Merck Group, Darmastad, Germany).

### 2.7. Antioxidant Activity Assay

The antioxidant activity was determined by two different assays: scavenging of DPPH (2,2-diphenyl-1-picryl-hydrazyl-hydrate) free radicals and TEAC (Trolox equivalent antioxidant capacity). The hydroalcoholic macerate of *P. caerulea* aerial parts was evaporated while using a Rotavapor^®^ R-300 (Büchi, Flawil, Switzerland), and the exact quantities were re-solubilized while using the recommended absolute alcohols. All of the assays were performed in triplicate. All of the reagents used were analytical-grade reagents purchased from Sigma–Aldrich (Merck Group).

#### 2.7.1. DPPH Scavenging Activity Assay

The DPPH scavenging activity was measured while using the method that was described by Huang et al. [51], with slight modifications: 150 μL DPPH methanolic solution (0.25 mM) was vigorously mixed with 15 µL of sample (resolubilized in methanol) and 90 µL of 0.1 M Tris-HCl buffer. The resulting mixture was incubated at 37 °C for 30 min. in the dark. Butylated hydroxytoluene (BHT) was used as a positive control. The sample absorbance (A_sample_) was read using a microplate reader (Sunrise, Tecan, Männedorf, Switzerland) at λ = 520 nm against a blank with methanol (A_blank_). DPPH inhibition (%) was calculated using the following equation:

% Inhibition = (1 − A_sample_/A_blank_) ∗ 100. (2)

#### 2.7.2. Antioxidant Capacity (TEAC) Assay

The antioxidant capacity (TEAC) was measured using the method of Re et al. [54], with slight modifications. The ABTS [(2,2’-azino-bis(3-ethylbenzothiazoline-6-sulphonic acid)] radical was generated by reacting a 7 mM 2,2’-azino-bis (3-ethyl-benzothiazoline-6-sulfonic acid) diammonium salt (ABTS) solution with a 2.45 mM potassium persulfate solution (1:1, *v*/*v*). The mixture was incubated in the dark at room temperature for 16 h. The initial absorbance of the ABTS radical solution was equilibrated to a value of 0.7 ± 0.02 at λ = 734 nm. Next, 0.1 mL test sample was mixed with 1 mL of the ABTS radical solution and incubated for 6 min. After incubation, the absorbance was measured at λ = 734 nm. A calibration curve of Trolox (0–250 μM) was used to convert the absorbance into the equivalent activity of Trolox per mL sample (µg Trolox/mL).

### 2.8. Cell Culture Biocompatibility

The in vitro biocompatibility tests were done on dried macerate (the hydroalcoholic extracts, vacuum dried, on a Rotavapor^®^ R-300, Büchi) that was resuspended into phosphate saline buffer (PBS). The resulting PBS extracts of the *P. caerulea* leaves were sterilized by filtration on 0.22 μm Millipore filters (Merck Millipore, Darmstadt, Germany). The in vitro biocompatibility assays for *Passiflora* extract were done while using a stabilized line of mouse fibroblast L929 cells (ATCC, cell line, NCTC clone 929), which was provided by the European Collection of Cell Cultures (ECACC). The NCTC L-929 cells were cultivated in Eagle’s MEM that was supplemented with 10% fetal bovine serum (FBS) and antibiotics (100 mg/mL penicillin, 100 mg/L streptomycin, and 500 mg/L neomycin) at 37 °C in a humidified incubator with 5% CO_2_ (CelCulture^®^ Incubator, Esco Medical, Barnsley, UK). The NCTC L-929 cells were seeded in 24-well plates at a cell density of 5 × 10^4^ cells/mL for 24 h to allow for adherence and were then incubated in the presence of different dilutions (1, 10, 50, 100, 150 µg/mL) of *P. caerulea* extracts for 24 and 48 h. The cell viability was determined using a colorimetric method, which was based on the ability of viable cells to incorporate a supravital dye, Neutral Red, NR [80]. Briefly, after the removal of the plant extract from the wells, a solution of the NR (50 mg/mL) was prepared in MEM that was supplemented with 10% fetal bovine serum. After an incubation period of 3 h at 37 °C in 5% CO_2_ atmosphere, the NR solution was removed, and an equal volume of fixative solution was added. The absorbance of the solution in the wells was read at λ = 540 nm while using a plate reader, i.e., Mithras LB 940 (Berthold Technologies, Bad Wildbad, Germany). The results were reported as percent viability relative to the control sample (cells incubated without the plant extract) considered 100% viable. The cell morphology was also evaluated. The NCTC L-929 cells, which were cultivated in the presence of various dilutions of plant extracts for 48 h, were fixed in methanol and then stained with a Giemsa solution for 20 min. Finally, the stained cells from different experimental treatments were examined under an optical microscope (Zeiss Observer, Carl Zeiss, Oberkochen, Germania), with a D1 20× objective. The dry weight (d_w_) of the tested *P. caerulea* extracts was determined by using a thermobalance (Moisture Analyzer Balance, Radwag, Cracow, Poland). The presented data are the average of three determinations. All of the reagents were suitable for cell culture and purchased from the Sigma–Merck Group.

### 2.9. Statistical Analysis

The data were statistically analyzed by analysis of variance (ANOVA) while using the SPSS 21 software package (IBM, Armonk, NY, USA). The data were analyzed in evolution, deducting the initial data from the treatment data before the application of the treatment. A least significant difference (LSD) test was used to separate the treatment means within each measured parameter at a significance level of *p* < 0.05. Coefficient k, from Equation (1), was statistically analyzed by the χ^2^ test (chi test).

## 3. Results

### 3.1. Effects on Morphological and Physiological Characteristics

**Figure 1:**
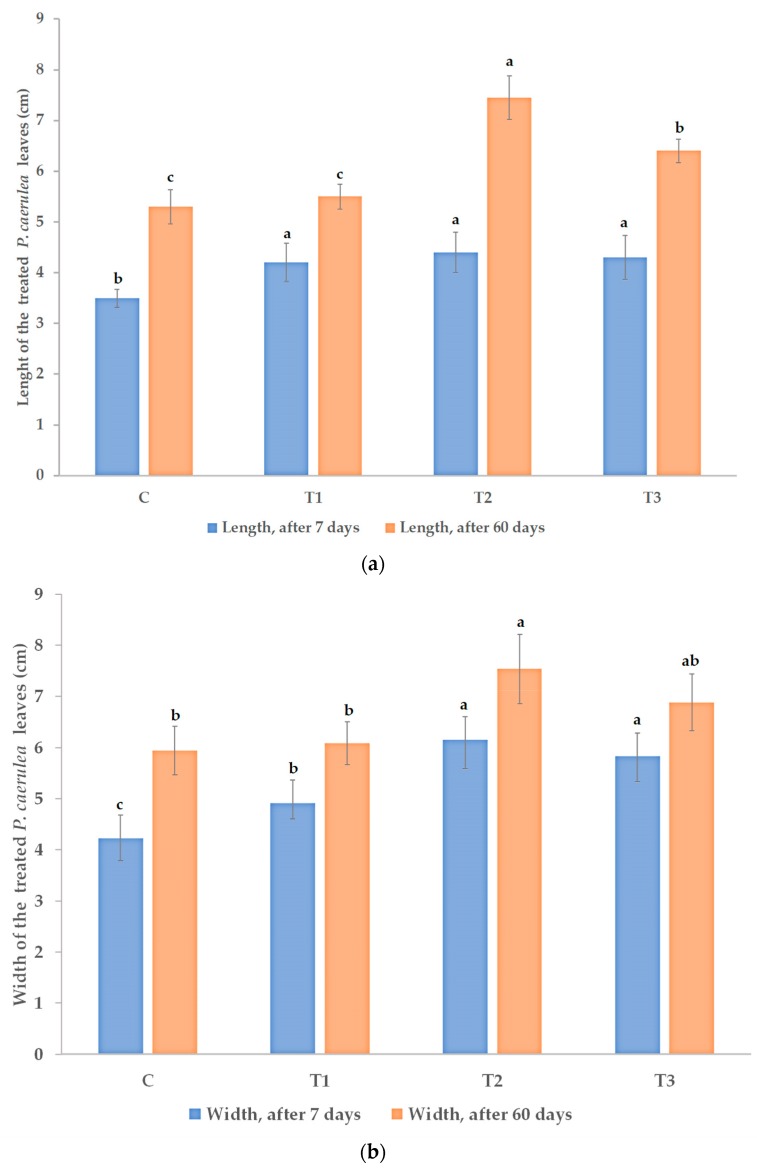

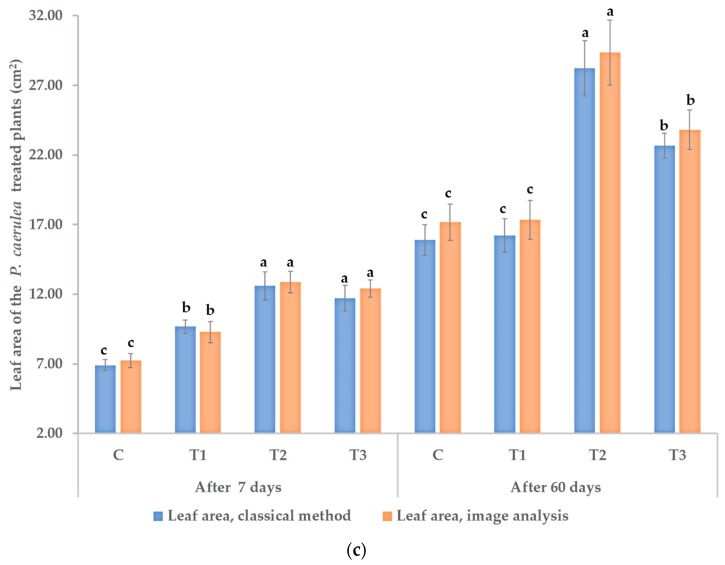
Effect of foliar treatment with plant biostimulants on the development of the leaves of *P. caerulea* plants after seven days from the first treatment and after 60 days from the first treatment: (**a**) effect on the leaf length (average of 10 leaves per replicate, four replicates per each treatment); (**b**) effect on the leaf width (average of 10 leaves per replicate, four replicates per each treatment); (**c**) effect on the average leaf area (cm^2^, average of 10 leaves per replicate, four replicates per each treatment). C—control (no treatment with plant biostimulants, treated only with water); T_1_—foliar treatment with *Trichoderma* consortium suspension, 10^6^ cfu/mL, equiv. to 10^11^ spores/ha; T_2_—foliar treatment with *Trichoderma* consortium suspension, 10^8^ cfu/mL, equiv. to 10^13^ spores/ha; T_3_—foliar treatment with a reference product, consisting of plant extracts, essential oils, and fatty acids in an oil/water emulsion and extract of the seaweed *Ascophyllum nodosum*, equiv. to 3 L/ha. The values presented represent means  ±  standard errors (*n*  =  40 plants). Columns that are labeled with different letters, compared between the different treatments (C, T1, T2, and T3) within each parameter and time period (after 7 days and, respectively, after 60 days) are significantly different at *p* < 0.05.

The effects of treatment with the *Trichoderma* consortium depended on the applied concentration. At the higher concentration, the stimulation of the foliar development lasted up to 60 days after the first treatment. Initially, after the first seven days, the boosting effect of the *Trichoderma* application, on the foliar development when compared to the water treated control was statistically significant for both spore concentrations. In the case of the higher spore concentration, the stimulation of leaf development was higher than 23% for the length of the leaf and almost 45% for the width of the leaf, when compared to the water-treated control, as in Figure 1a,b.

The initial timepoint measurement (at seven days after the first treatment) was intended to determine the capacity of the lower concentration of *Trichoderma* consortium to colonize a small habitat. As mentioned, both concentrations had a significant stimulatory effect at seven days after the first treatment, when compared to water treated control. However, lower concentration of *Trichoderma* consortium determined lower stimulation of leaves development in length (around 20% stimulation) and width (around 15% stimulation). After 60 days, the leaf development was significantly stimulated only by the highest applied concentration of the *Trichoderma* consortium—more than 40% stimulation of leaf length and around 40% stimulation of the width. The reference plant biostimulant product, which was based on natural extracts, including volatile compounds (essential oils) and elicitors (from algae and plant extracts), had an intermediate influence under our experimental conditions, with a more pronounced stimulation effect after the second treatment when compared to the first week—Figure 1a,b. The development of leaves increases the dimensions of the habitat to be colonized by the applied microbial consortium. High leaf surface reduces the chances of the lower concentrated *Trichoderma* consortium spores to successfully colonize the enlarged habitat.

These longer lasting effects of the *Trichoderma* consortium applied at 10^8^ cfu/mL concentration and, partially, of the reference product on leaf morphology also determined an increase in the leaf area, as in Figure 1c. The differences between the classical method of calculation of leaf area and the image analysis method were less than 5% (Figure 1c). However, we used two different modes of calculation, because the classical method of leaf area calculation objectively evaluates the leaf irregularities. Within this formula, the coefficient k, which is specific to different plant species/varieties and plant development stages, is used. The average value that was calculated for coefficient k was 0.47 at the beginning of the experiment and 0.53 at the end of the experiment. The distribution of the coefficient k, being statistically analyzed by the χ^2^ (Hi/chi squared) test, did not vary between treatments. These data are in agreement with the already published data [75], which established the specificity of coefficient k for *Passiflora* spp., confirming the values that were calculated (k = 0.47–0.53) as being specific for a plant with normal development. The small differences between the two methods for calculation of the leaf area and the calculated k value, within the range of the published coefficient, demonstrate that the stimulation of *P. caerulea* by the *Trichoderma* consortium applied at the higher rate was not associated to the deviation of the leaves from their normal development.

**Figure 2:**
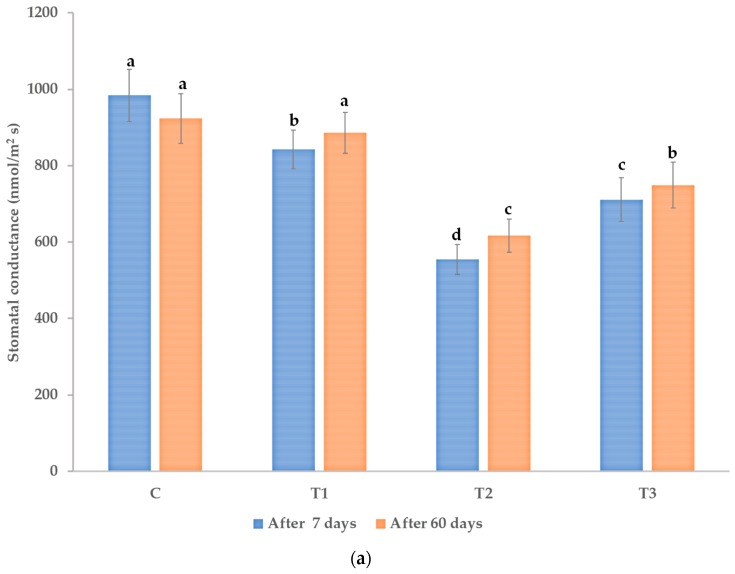

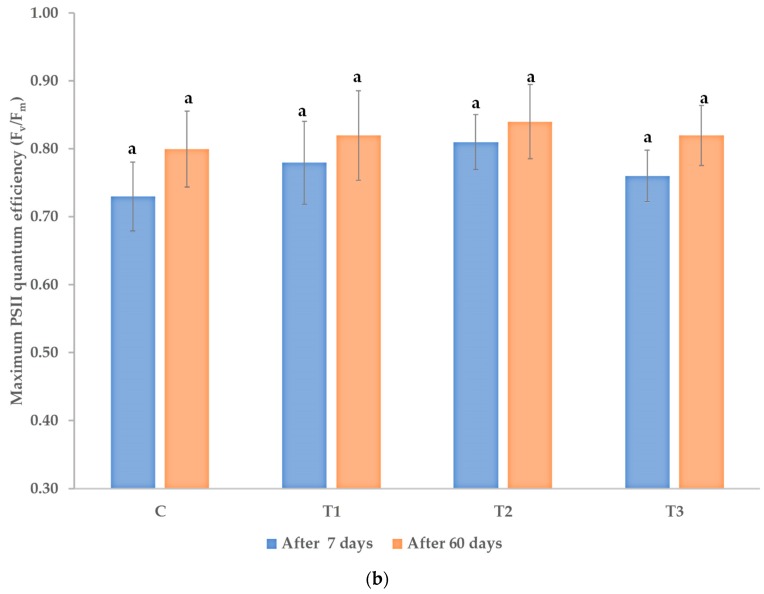
(**a**) Stomatal conductance (nmol/m^2^ s) and (**b**) maximum PSII quantum efficiency of *P. caerulea* plants after seven days from the first treatment and after 60 days from the first treatment with plant biostimulants. C—control (no treatment with plant biostimulants, treated only with water); T_1_—foliar treatment with *Trichoderma* consortium suspension, 10^6^ cfu/mL, equiv. to 10^11^ spores/ha; T_2_—foliar treatment with *Trichoderma* consortium suspension, 10^8^ cfu/mL, equiv. to 10^13^ spores/ha; T_3_—foliar treatment with the reference product, consisting of plant extracts, essential oils and fatty acids in an oil/water emulsion, and extract of the seaweed *Ascophyllum nodosum*, equiv. to 3 L/ha. The values presented are means  ±  standard error (*n*  =  40 plants). Columns labeled with different letters, compared between the different treatments (C, T1, T2, and T3) within each time period (after 7 days and, respectively, after 60 days) are significantly different at *p* < 0.05.

Our determinations (Figure 2a) demonstrate a lower stomatal conductance for plant biostimulant-treated plants compared to the control. The *Trichoderma* treatments were previously shown to reduce stomatal aperture and improve the water use efficiency of the model plant *Arabidopsis thaliana* [81]. We observed a similar effect on *P. caerulea* under our experimental conditions—Figure 2a.

We estimated the maximum quantum efficiency of PSII by *in situ* determination of the chlorophyll fluorescence of the pre-darkened leaves. Our measurements on the *Passiflora* leaves do not demonstrate a statistically significant activation of the photosynthetic efficiency following plant biostimulant treatment, as in Figure 2b. *Trichoderma* plant biostimulants were reported to enhance the intensity of photosynthesis and to modulate chlorophyll fluorescence in Micro-Tom tomatoes [82]. Under our experimental conditions, with measurements done under high photosynthetic active radiations, we only noticed a tendency for an increased photosynthetic efficiency, which is at the limit of statistical significance of *p* < 0.05.

### 3.2. Effects on Leaf Ultrastructure

**Figure 3:**
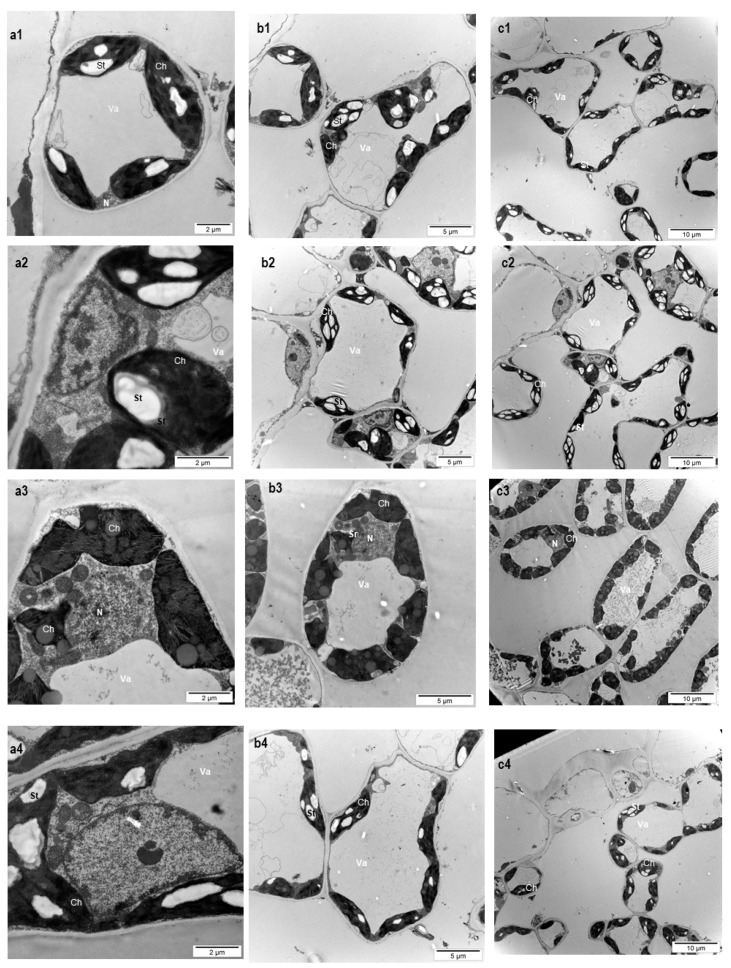
Ultrastructural aspects of the spongy parenchyma cells of *P. caerulea* leaves after 60 days from the first treatment with plant biostimulants; (**a1**,**b1**,**c1**): C, control (no treatment with plant biostimulants, treated only with water); (**a2**,**b2**,**c2**): T_1_, foliar treatment with *Trichoderma* consortium suspension, 10^6^ cfu/mL, equiv. to 10^11^ spores/ha; (**a3**,**b3**,**c3**): T_2_—foliar treatment with *Trichoderma* consortium suspension, 10^8^ cfu/mL, equiv. to 10^13^ spores/ha; (**a4**,**b4**,**c4**): T_3_, foliar treatment with a reference product, consisting of plant extracts, essential oils and fatty acids in an oil/water emulsion, and extract of the seaweed *Ascophyllum nodosum*, equiv. to 3 L/ha. Ch—chloroplast; Va—Vacuole; St—Starch grains; N—nucleus; **a**,**b**,**c**—different magnifications.

The plant treatment with the *Trichoderma* consortium at the higher concentration resulted in an increased number of chloroplasts in spongy parenchyma, with larger surface when compared to the control—Table 1, Figure 3.

Following application of the higher concentration of *Trichoderma* consortium spores, the number of chloroplasts increased by 62.02% and the surface of the chloroplasts almost doubled—an increase of 91.66%. The chloroplast enlargement was not statistically significant in the case of the leaves that were subjected to treatment with the lower amount of the *Trichoderma* consortium or with the reference product. In the case of the lower *Trichoderma* consortium concentration, the overall anatomy of the spongy parenchyma cells tissue was not significantly changed. The chloroplast number and surface did not increase significantly. The treatment with the reference product, wherein volatile compounds and elicitors are included, tended to change the chloroplast number and the chloroplast surface from the cell spongy parenchyma cells. However, this tendency is not statistically significant. The analyzed spongy parenchyma cells ultrastructure suggests that the application of the *Trichoderma* consortium at the concentration of 10^8^ ufc/mL leads to successful colonization of *P. caerulea* phyllosphere.

### 3.3. Polyphenols, Flavonoids, and Antioxidant Activity in Leaves of the Treated Plants

**Figure 4:**
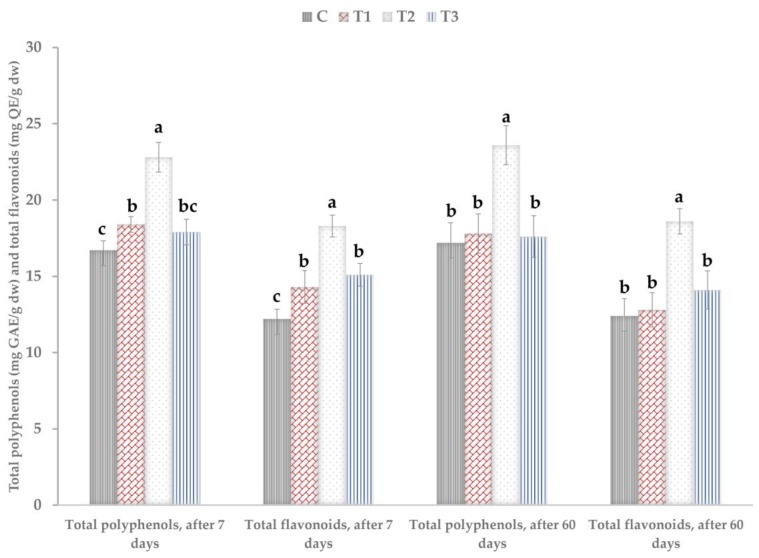
Total polyphenols (mg GAE/g dw) and total flavonoids (mg QE/g dw) in the hydroalcoholic extracts from aerial parts of the *P. caerulea* plants after seven days from the first treatment and after 60 days from the first treatment with plant biostimulants. C—control (no treatment with plant biostimulants, treatment only with water); T_1_—foliar treatment with *Trichoderma* consortium suspension, 10^6^ cfu/mL, equiv. to 10^11^ spores/ha; T_2_—foliar treatment with *Trichoderma* consortium suspension, 10^8^ cfu/mL, equiv. to 10^13^ spores/ha; T_3_—foliar treatment with a reference product, consisting of plant extracts, essential oils, and fatty acids in an oil/water emulsion, and extract of the seaweed *Ascophyllum nodosum*, equiv. to 3 L/ha. Values presented are means  ±  standard error (*n*  =  12 replicates). Columns labeled with different letters within each parameter are significantly different at *p* < 0.05.

The marketable yield of the *P. caerulea* nutraceutical crop is represented by the aerial parts, which can be harvested without compromising the development of the plants in the next year. Maceration in Et(OH)–water 70% (*v*/*v*) solution was chosen for the extraction of polyphenols and flavonoids from dried and ground aerial parts of *P. caerulea*, in accordance with our previous work, in which we found that this method recovered the highest concentration of polyphenols and flavonoids from this passion plant material [73]. The treatments with the plant biostimulants promoted the accumulation of polyphenols and flavonoids into the aerial parts of *P. caerulea* plants, as in Figure 4.

The accumulation of both polyphenols and flavonoids was most significant in plants that were treated with the higher concentration of the *Trichoderma* plant biostimulant consortium, 10^8^ cfu/mL, more than 35% when compared to the control for both sampled periods (7 days and 60 days after the first treatment). In the case of the lower concentration of the *Trichoderma* consortium, the accumulation of higher quantities of polyphenols and flavonoids was not statistically significant after 60 days from the first treatment. However, for the plant material that was sampled after seven days, the application of the lower concentration of the *Trichoderma* consortium resulted in a significant increase in the total polyphenols and flavonoids—by more than 10% higher than the water treated control. The reference product promoted a significant accumulation of total flavonoids after the first treatment—an almost 24% increase of the total flavonoids when compared to the control. The effect resulting from the reference plant biostimulant was less significant after 60 days, when the increase in the total flavonoids was around 14%.

**Figure 5:**
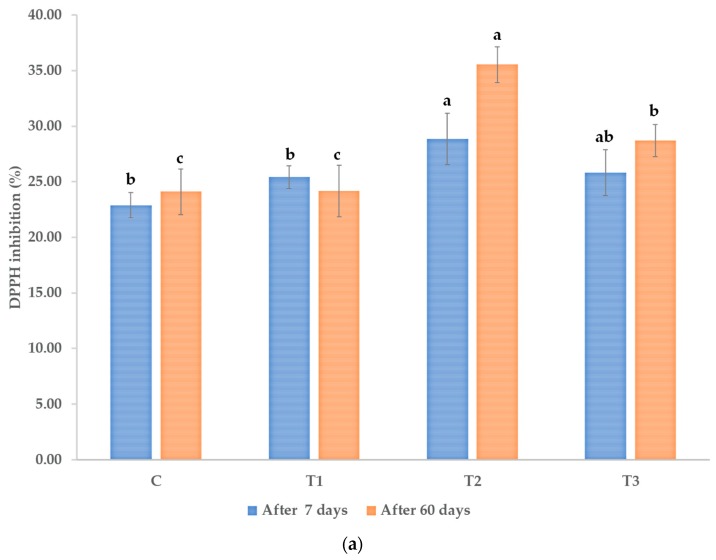

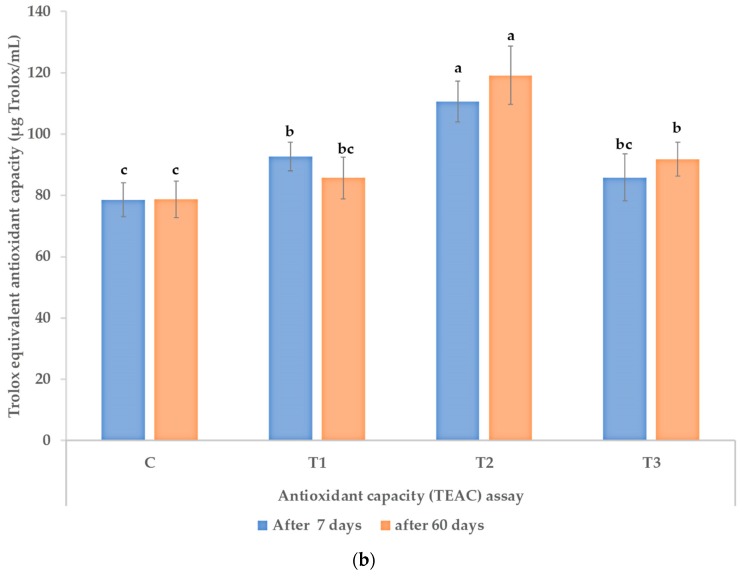
Antioxidant activity of the hydroalcoholic extract of aerial parts of the *P. caerulea* plants, determined as the ability to scavenge the formation of the DPPH (2,2-diphenyl-1-picryl-hydrazyl-hydrate) free radicals (**a**) and TEAC (Trolox equivalent antioxidant capacity) (**b**), after seven days from the first treatment and after 60 days from the first treatment. C—control (no treatment with plant biostimulants, treatment only with water); T_1_—foliar treatment with the *Trichoderma* consortium suspension, 10^6^ cfu/mL, equiv. to 10^11^ spores/ha; T_2_—foliar treatment with the *Trichoderma* consortium suspension, 10^8^ cfu/mL, equiv. to 10^13^ spores/ha; T_3_—foliar treatment with a reference product, consisting of plant extracts, essential oils and fatty acids in an oil/water emulsion, and extract of the seaweed *Ascophyllum nodosum*, equiv. to 3 L/ha. The values presented are means  ±  standard error (*n*  =  12 replicates). Columns labeled with different letters, compared between the different treatments (C, T1, T2, and T3) within each time period (after 7 days and, respectively, after 60 days) are significantly different at *p* < 0.05.

A similar effect was observed for the antioxidant activity, as in Figure 5a,b. The highest concentration of the *Trichoderma* plant biostimulant consortium, 10^8^ cfu/mL, resulted in the highest antioxidant activity in both assays (DPPH and TEAC).

After seven days from the first treatment, the antioxidant activities of the hydroalcoholic extracts from passion plants that were treated with *Trichoderma,* 10^8^ cfu/mL were 26.12% and 40.35% higher than the control, as determined by the DPPH method and the ABTS/TEAC method, respectively. After 60 days from the first treatment, the increase became more pronounced in the case of the DPPH assay, 47.37% higher activity of extracts from plants treated with *Trichoderma,* 10^8^ cfu/mL, than the control. For the TEAC assay, the increase as compared to the control exceeded 50%. The effects of the lower concentration of *Trichoderma* plant biostimulant treatment, 10^6^ cfu/mL, were only statistically significant in the case of the plant material sampled after seven days from the first treatment. In the case of the antioxidant activity determined by the TEAC assay, the activity of the lower concentration was 17.93% higher than the activity of the extracts from the control plants after seven days. Treatment with the reference products containing elicitors from plants and seaweeds resulted in a significant increase in the antioxidant activity that was assayed by the DPPH method, at both timepoints (12.84% at seven days and 19.07% at 60 days after the first treatment)). In the case of the TEAC method the increase was significant only at 60 days from the first treatment—16.63% as compared to the control.

### 3.4. Cytocompatibility of the Extracts from the Treated Leaves

The stimulation of bioactive compounds accumulation in *P. caerulea* leaves, especially of polyphenols and flavonoids, could lead to combinations with reduced safety [83]. High doses of polyphenols proved to be toxic in some cases [84]; therefore, a screening of the safety of treated plant extracts was considered to be necessary. An *in vitro* method based on cultured cell viability assay was selected for safety screening of the blue passion flower extracts. Such techniques, corresponding to the reduction–replacement-refinement ethical principles of laboratory animal tests, offer results that are consistent with ingestion/*in vivo* studies [85] and also have the advantages of rapidity and lower costs when compared to the *in vivo* techniques [86].

**Figure 6:**
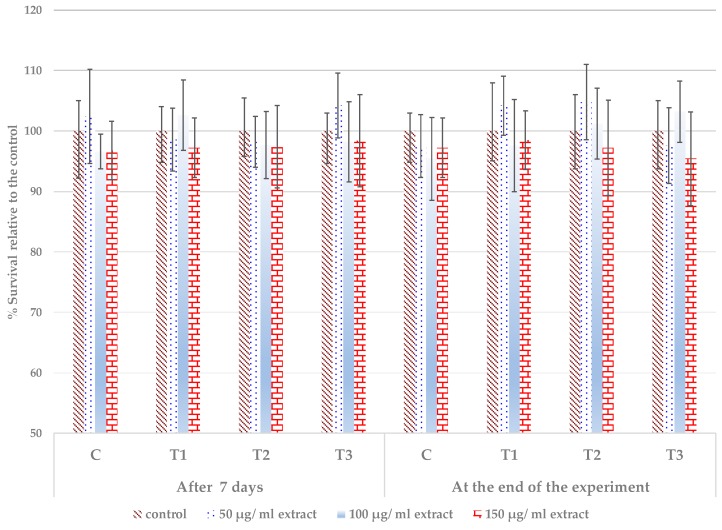
Mouse fibroblast NCTC L929 cell viability after incubation for 48 h with different concentrations of a hydroalcoholic extract of aerial parts of the *P. caerulea* plants sampled after seven days from the first treatment and after 60 days from the first treatment with plant biostimulants. C —plant control (no treatment with plant biostimulants, treated only with water); T1—foliar treatment with the *Trichoderma* consortium suspension, 10^6^ cfu/mL, equiv. to 10^11^ spores/ha; T2—foliar treatment with the *Trichoderma* consortium suspension, 10^8^ cfu/mL, equiv. to 10^13^ spores/ha; T3—foliar treatment with a reference product, consisting of plant extracts, essential oils, and fatty acids in an oil/water emulsion, and the extract of the seaweed *Ascophyllum nodosum*, equiv. to 3 L/ha. The values presented are means  ±  standard errors (*n*  =  12 replicates) and are not statistically different.

The *in vitro* biocompatibility of the treated leaf extracts was assessed on a stabilized line of mouse fibroblast NCTC L929 cells, while using the MTT (dimethylthiazol-diphenyltetrazolium bromide) assay. This colorimetric assay evaluates the activity of mitochondrial succinate dehydrogenase (which reduces the soluble MTT tetrazolium salt to the insoluble blue MTT formazan product) as a marker of cell viability. The results that were obtained after 48 h of cell incubation did not demonstrate the presence of cytotoxic compounds. All of the tested extracts, which were obtained from *P. caerulea*-treated leaves, proved to be compatible with the mouse fibroblast NCTC L929 cell culture in the tested concentration range of 50–150 μg/mL, as in Figure 6. The pattern of cytocompatibility shows some differences between the plant materials, depending on the sampling date, treatment, and concentration applied. At low (50 μg/mL) and intermediate (100 μg/mL) concentration tested, some of the variants induced a slight stimulation of the viability (low dose – C and T3 sampled at 7 days, T2 and T3 sampled at 60 days; intermediate dose – T2 at 7 days, T3 and T4 at 60 days). Other variants induced a slight decrease in the viability (low dose – C and T1 at 7 days, C and T3 at 60 days; intermediate dose – C, T2 and T3 at seven days, T1 at 60 days). The highest tested concentration of 150 μg/mL slightly reduced the cell viability for all of the tested extracts from all treatments.

However, these differences were not significant. Usually, a tested sample is considered to be cytotoxic/not cytocompatible when the viability is reduced to less than 80% [87,88]. The cell morphology was normal, without alteration of the cell membrane structure—Supplementary Material, Figure S1. This finding supports the good cytocompatibility of the extracts from the treated *P. caerulea* leaves.

### 3.5. Effects on Yield and Yield Quality

The main aim of treatment with the *Trichoderma* plant biostimulant consortium was to increase the yield and improve the yield quality following the successful colonization of the phyllosphere by the applied plant beneficial strains. The treatments with the *Trichoderma* consortium at the higher spore concentration resulted in a better development of the passion plant canopy, with higher leaf surface, increased biomass accumulation, and increased marketable yield. Together with an increased biomass accumulation, a higher level of bioactive compounds accumulated in the aerial parts of the treated plants. At 60 days after the first treatment, the production of the human health-promoting compounds in the *P. caerulea* nutraceutical crop was significantly increased by the application of the *Trichoderma* plant biostimulant consortium at a concentration of 10^8^ cfu/mL. In Table 2, we summarized the marketable yield of the treated nutraceutical plants, integrating the quality features (total polyphenols and total flavonoids) with the marketable yield—i.e., the quantity of the leaves that could be harvested without compromising the *P. caerulea* perennial plant future development.

The accumulation of the total polyphenols and flavonoids was 75% and, respectively, more than 90% higher in the marketable yield when compared to the water-treated control. The difference between the effects that were produced in the passion plants by the two concentrations of the *Trichoderma* plant biostimulant consortium was evident for the leaf growth rate, which was significantly higher at the higher spore concentration, 10^8^ cfu/mL, than at the lower spore concentration, 10^6^ cfu/mL, as shown above (Figure 1c). A high growth rate of the treated leaves was demonstrated for the treatment with the reference plant biostimulant product—this effect is probably related to both active ingredients from seaweeds/plant extracts and the micro-nutrient contents in this reference biostimulant. The higher leaf growth rate determines the marketable biomass yield, which was the highest for the higher concentration of *Trichoderma* and for the reference products. In plants treated with the *Trichoderma* consortium (10^8^ cfu/mL), the dried weight of the harvestable leaves increased by more than 27% as compared to the control treated with water and reached 1.15 Kg of dried plant material for 15 harvested plants, as in Table 2. The significant increase of polyphenols and flavonoids accumulation following treatment with the *Trichoderma* consortium at the higher concentration determined a higher yield of the bioactive compounds in the marketable production.

## 4. Discussion

Despite the fact that the *Trichoderma* strains are generally considered to be specific to soil and plant root systems—the rhizosphere and/or rhizoplane habitat—efficient strains have been used for decades as biological control agents in the phyllosphere, the microbial habitat that is specific to the aerial parts of the plants [89]. Phyllosphere/phylloplane fungi are challenged by the much lower nutrient availability as compared to the rhizosphere and by higher abiotic stress—i.e., moisture, temperature, higher temperature and humidity variations, and ultra-violet light. However, multifunctional/multifaceted strains, which originate from the rhizosphere, such as those that are used in this study, have been proven to have plant biostimulant effects after application as foliar treatment. *T. harzianum* T22, a strain that is largely used for the production of commercial bioproducts, originating from soil and well-known for its rhizosphere competence [10], was applied for the treatment of the grape phyllosphere [35]. A volatile secondary metabolite, 6-pentyl-2H-pyran-2-one—6-PP, specific to *Trichoderma*, was also applied as a reference for the T22 treatment. Repeated applications of foliar treatments with the T22 strain (10^8^ spores/liter) and 6-PP (1 µM solution) resulted in increased crop yield, enhanced the accumulation of polyphenols, and induced higher antioxidant activity. 6-PP was more active in inducing the antioxidant activity in raisins (+60.3%) when compared to T-22—48.7%.

In the above-mentioned study, repeated treatment was the strategy used to promote the establishment of the applied strain in the phyllosphere habitat. Other successful strategies for promoting the colonization of the phyllosphere/phylloplane by inoculated strains include the manipulation of the abiotic conditions, i.e., humidity [90], inclusion into controlled released formulations, which specifically release propagules into the leaf environment [91], and the application of higher propagule density [92]. In this study, we used the strategy related to a higher density of the initial inoculum, while only using the spores (with a high proportion of chlamydospores) that were collected after cultivation on specific media [68,69] and that survive well in the environment [93]. A concentration of 10^8^ spores/mL, 1000-fold higher than that used for T22 applied on grape [35], causes a long-lasting effect and a significant increase in bioactive compounds in *P. caerulea*. The lower concentration applied, 10^6^ spores, probably does not ensure enough inoculum for the *Trichoderma* to compete and colonize the phyllosphere niche, especially when the canopy of the passion plant increases due to leaf development. In small leaves, with small surface, the lower concentration of the applied spores also has a significant effect (at seven days after the first treatment). However, after the second treatment, when the surface of the leaf increases, the applied lower concentration of the *Trichoderma* consortium does not continue to influence the treated plants, most probably due to the failure of colonization of the phyllosphere.

The colonization of the leaves by the *Trichoderma* consortium was associated with enhanced growth of the *P. caerulea* plants. As previously mentioned, both strains used for the applied *Trichoderma* consortium produce bioactive volatile compounds, including 6-PP [62,63], are antagonists of major plant pathogens [63,64], protect and stimulate vegetable growth [65], and produce enzymes and other active molecules (e.g., cerato-platanin) that promote the degradation of lignocellulose material [66,67]—microbial elicitors (pathogen or microbial-associated molecular patterns—PAMPs, or, respectively, MAMPs) [13,14]. PAMPs and MAMPs trigger the innate immunity—PTI (PAMPs-triggered immunity) and MTI (MAMPs triggered immunity) [94]. *Trichoderma* is considered to be a model to study these microbial effectors of the innate immunity/plant defence [95].

Chloroplasts play an essential role in plant innate immunity at the cellular level [96,97]. These organelles are not involved only in the primary metabolism, such as light energy harvester or for photosynthesis of carbohydrates, but are also the sites for the biosynthesis and transmission of signals that are involved in plant defence/innate immunity responses [98]. To the best of our knowledge, there are no reports that relate *Trichoderma* triggered immunity to the ultrastructure or functions of chloroplasts, despite the fact that *Trichoderma* is a model for plant defence/innate immunity triggering [95]. Very recently, it was communicated that L-amino Acid Oxidase secreted by *Trichoderma* triggers the innate immunity by targeting Chlorophyll a/b Binding Proteins from chloroplasts [99]. Our ultrastructural analysis of the spongy parenchyma demonstrates an increase in the number and surface/volume of the chloroplasts from treated plants. Such finding suggests that the chloroplasts play a role in the plant defense activation exerted by *Trichoderma*, which gives a new perspective of the well-known enhancement of the photosynthetic capacity that is determined by *Trichoderma* treatments. The promotion of chloroplast development in the case of treatment with the higher concentration of *Trichoderma* spores is another indirect evidence, besides the morpho-physiological features, of the successful colonization of the phyllosphere promoted by the higher applied concentration.

The environmental factors are important for the effects of plant biostimulants. By definition, plant biostimulants enhance plant tolerance to abiotic stress [100]. In horticultural crops, plant biostimulants are considered to be important agronomic tool to mitigate environmental/abiotic stress effects [101]. Therefore, experimental field tests of plant biostimulants must be done with plants that have been subjected to environmental/abiotic stress conditions. The main stress for the *P. caerulea* plants which we used in our field experiments is related to light intensity. *P. caerulea*, as the other plants from *Passiflora* genera, is a liana, a woody vine that climbs on trees and develops under shade conditions. Passion plants are cultivated as shrubs, in full sunlight. However, the optimal photosynthetic radiation for *Passiflora* plants was shown to be ~25% (300–650 μmol m^−2^ s^−1^) or ~50% (400–750 μmol m^−2^ s^−1^) from the corresponding photosynthetically active radiation (PAR) that is specific to full sunlight (1000–1500 μmol m^−2^ s^−1^, minimum–maximum) [102]. The light intensity stress could also explain our findings that are related to the lack of effect of the plant biostimulant treatments on the photosynthetic efficiency determined by the chlorophyll fluorescence assay.

Light intensity stress in plants is associated with higher production of reactive oxygen species (ROS) [103]. It was shown that *Trichoderma* treatments reduce the effects that are produced on plants by various biotic or abiotic stresses by the modulation of the reactive oxygen species and reduction of their accumulation at pathological level [104,105,106]. A similar mechanism, together with the increase in the number and surface of chloroplasts, could be probably involved in our study, the treatment inducing protection of the blue passion plant against high light intensity by a reduction of accumulation of reactive oxygen species. It was shown that the ultrastructure of chloroplasts (including number and surface) is a reliable marker for stress, including light stress [76]. Therefore, the increased number of larger chloroplasts could be the result of efficient light intensity stress mitigation by the *Trichoderma* consortium that is established within the leaf habitat, following the application of the higher concentration of the more resistant chlamydospores.

Treatments with *Trichoderma* are well-known to increase the photosynthetic plant capability [24]. Our focus on the ultrastructure of the spongy parenchyma cells was motivated by their important function in photosynthesis, during both light and dark phases. Such cells provide uniformity in light absorption by the leaves [107]. Due to their organization, with spaces on the top of the lower epidermis (wherein more stomata are usually located), spongy parenchyma cells are more efficient in performing dark phase photosynthesis-CO_2_ fixation [108]. Additionally, the spongy parenchyma tissue porosity is essential for mesophyll diffusion conductance to CO_2_, being recognized at present as being a major limiting factor for photosynthesis and for photosynthetic resource use efficiency [109]. Recent studies demonstrated that spongy parenchyma cells are also photosynthetically active under UV stress [110].

In a previous study, we tested the influence of the treatments with the same *Trichoderma* consortium, applied at 10^8^ cfu/mL, on *P. caerulea* leaf development and leaf morpho-anatomical features. We reported that the mesophyll from the treated plants is more voluminous, with both palisade and spongy parenchyma cells being more developed and with larger plant cell walls [72]. In the present study, we investigated the changes in the ultrastructural features and established the importance of spore concentration for a successful long-term phyllosphere colonization, together with other aspects, such as the yield and yield quality.

The ultrastructural analysis of spongy parenchyma cells confirmed our previous light microscopy determinations. It reveals that the *Trichoderma* treatment at 10^8^ cfu/mL determines not only an increase in the number and surface of the chloroplasts, but also visibly reduces the volume and number of the starch grains (Figure 3). The reduction of chloroplast starch grains suggests a more efficient translocation of photosynthesized carbohydrates from chloroplasts to the rest of the cell and/or to the phloem. Various stresses, such as high temperature [111], salt stress and gamma rays [112], or heavy metal stress [113], were reported to generate large starch grains in the chloroplasts, due to the disruption of the photosynthesized carbohydrates translocation.

The yield quality is also improved in the *Trichoderma*–*Passiflora* interaction, when the *Trichoderma* consortium is applied at higher spore rate. The bioactive compounds that accumulate in the passion plants that are treated with the *Trichoderma* consortium are polyphenols and flavonoids. This is important for the yield quality of *P. caerulea*—we have already presented the involvement of the polyphenols and flavonoids as the main active ingredients that are responsible for the effects of passion flowers on human health and wellbeing [49,50].

Enhanced polyphenols accumulation suggests the activation of their main biosynthesis pathway in plants, the phenylpropanoid pathway [114]. Recently, it was demonstrated that the *Trichoderma* treatment activates this pathway [115]. The enlargement of the plant cell wall under the effect of treatment with *Trichoderma* consortium spores, which we previously reported [72], is an indirect proof of the phenylpropanoid pathway activation and it is directly associated with polyphenol accumulation. The phenylpropanoid pathway is connected to lignin biosynthesis, and lignin is one of the main components of the plant cell wall [116]. The phenylpropanoid pathway association with the shikimate and the quinate pathways also cause the accumulation of hydroxycinnamic acids [117], polyphenols that anchor the hydrophobic and hydrophilic parts of the plant cell wall [118]. Therefore, the activation of the phenylpropanoid pathway leads to the accumulation of polyphenols, cell wall proliferation, and enhanced plant resistance to biotic and abiotic stress.

The antioxidant activity of polyphenols and flavonoids has been related to health benefits [51,52,53]. In this study, we found a correlation between the accumulation of the antioxidant polyphenols and flavonoids and decreased stomatal conductance (a proxy for water use efficiency). Other studies demonstrated that antioxidant polyphenols/flavonoids are involved in enhanced tolerance to drought/water stress, tolerance that is induced by treatment with the plant biostimulant *Trichoderma* [20,119]. The effects are probably related to the cross-talk of the antioxidant bioactive compounds with the reactive oxygen species (ROS) and their signaling cascade [120]. The role of ROS quenching and homeostasis in the regulation of stomatal conductance and water use efficiency was recently highlighted [121]. The accumulation of polyphenols causes enhanced tolerance to abiotic stress [114]. Therefore, we conclude that the application of the *Trichoderma* consortium, which activates plant secondary metabolism and stimulates the accumulation of antioxidant polyphenols and flavonoids, also has an agronomic benefit, in addition to the effects on human-health promoting properties of crop products. In nutraceutical crops, such multi-level effects, which result from the interaction of the cultivated plants with multi-functional/plant biostimulant *Trichoderma* strains, have a significant economic effect, due to the combination of a higher yield and better quality/health-promoting properties.

## 5. Conclusions

The *Trichoderma* consortium formed by two strains from the INCDCP-ICECHIM collection, *T. asperellum* T36 NCAIM F 001434 and *T. harzianum*, Td50b NCAIM F001412, being applied at the concentration of 10^8^ cfu/mL, induced a significant long-term stimulation of leaf growth and of bioactive compounds accumulation. Plant yield and quality both increased after the *Trichoderma* consortium treatment. The observed effects on plant physiology and plant cell wall proliferation suggest enhanced resistance of the treated plants to stress. Applying a high rate of resistant *Trichoderma* strains spores (e.g., chlamydospores) seems to promote long-term phyllosphere colonization and could represent a more efficient strategy for obtaining higher crop yield and quality, by reducing the number of treatments.

## 6. Patents

A patent application, RO 131827 A2, was filled in relation to the *Trichoderma* consortium presented in this paper. This consortium, formed by two strains from the INCDCP-ICECHIM collection, *T. asperellum* T36 NCAIM F 001434 and *T. harzianum* Td50b NCAIM F001412, both being deposited at the National Collection of Agricultural and Industrial Microorganisms (NCAIM), is highly biocompatible and demonstrated synergic plant biostimulant effects on nutraceuticals and vegetable crops.

## Figures and Tables

**Table 1:** Number and surface (in µm^2^) of the chloroplasts from spongy parenchyma of *P. caerulea* plants treated with plant biostimulants.

Variant	Chloroplast Number (*n* = 100)	Chloroplast Surface, µm^2^ (*n* = 40)
C—control (no treatment with plant biostimulants, treated only with water)	7.9 ± 0.4b	4.8 ± 0.8b
T_1_—foliar treatment with *Trichoderma* consortium suspension, 10^6^ cfu/mL, equiv. to 10^11^ spores/ha	8.4 ± 0.3b	5.3 ± 0.6b
T_2_—foliar treatment with *Trichoderma* consortium suspension, 10^8^ cfu/mL, equiv. to 10^13^ spores/ha	12.8 ± 0.7a	9.2 ± 1.1a
T_3_—foliar treatment with a reference product, consisting of plant extracts, essential oils and fatty acids in an oil/water emulsion, and extract of the seaweed *Ascophyllum nodosum*, equiv. to 3 L/ha	8.9 ± 0.6b	6.2 ± 0.9b

Values presented are means  ±  standard error. Values followed by the same letter are statistically similar for a specific parameter, according to LSD (*p* < 0.05); *n* represents the number of cells investigated.

**Table 2:** Marketable dried yield of aerial plants, total polyphenols, and total flavonoids in the harvested biomass from the *P. caerulea* plants that were treated with the plant biostimulants.

No.	Treatment	Marketable Yield, Dried Weight (Kg/15 Plants)	Total Polyphenols Harvested (g/15 Plants)	Total Flavonoids Harvested (g/15 Plants)
C	Control (untreated)	0.90 ± 0.04b	15.48 ± 2.06b	11.16 ± 2.48b
T_1_	*Trichoderma* 10^6^ ufc/mL, equiv. 2 × 10^11^ spores/ha	0.92 ± 0.06b	16.37 ± 2.82b	11.77 ± 2.84b
T_2_	*Trichoderma* 10^8^ ufc equiv. 2 × 10^13^ spores/ha	1.15 ± 0.05a	27.14 ± 4.73a	21.39 ± 4.23a
T_3_	Reference product, plant oil, algae, and plant extract	1.01 ± 0.08ab	18.48 ± 4,27ab	14.24 ± 4.05b

The values presented are means ± standard errors (*n* = 4 replicates). Values followed by the same letter are statistically similar for a specific parameter, according to LSD (*p* < 0.05).

## Supplementary Materials

Supplementary materials can be found at https://www.mdpi.com/2076-2607/8/1/123/s1. Figure S1. The morphology of the mouse fibroblast NCTC L929 cells cultivated in media with treated plant extracts.
