# Supplementary material for: Effects of Foliar Treatment with a Trichoderma Plant Biostimulant Consortium on Passiflora caerulea L. Yield and Quality
Source: Microorganisms. 2020 Jan 16;8(1):123. doi: 10.3390/microorganisms8010123 (PMC7023023; doi:10.3390/microorganisms8010123)
Supplement: Supplementary file 1 [file microorganisms-08-00123-s001.pdf]

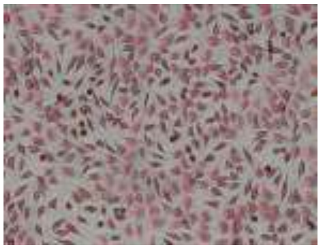

Control culture, without plant extract

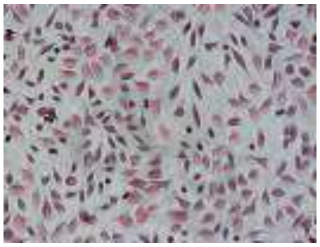

C - sampled 7 days after treatment

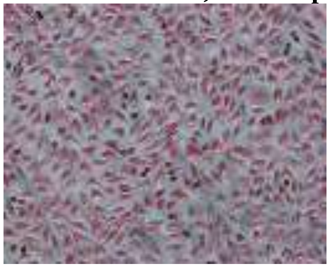

T_1 - sampled 7 days after treatment

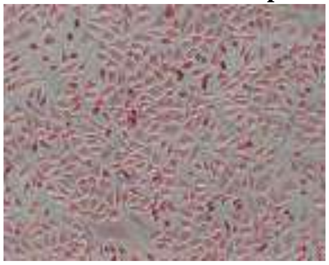

T_2 - sampled 7 days after treatment

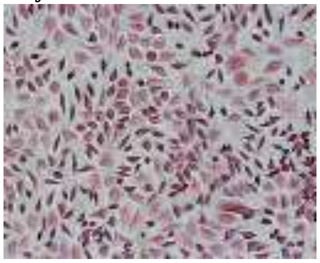

T_3 - sampled 7 days after treatment

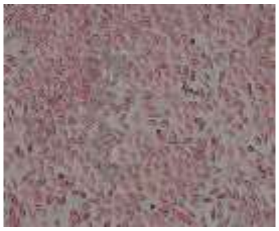

Control culture, without plant extract

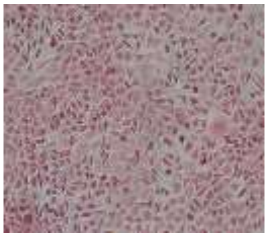

C - sampled after 60 days

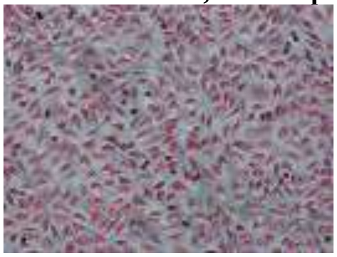

T_1 - sampled after 60 days

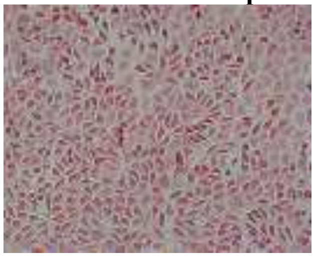

T_2 - sampled after 60 days

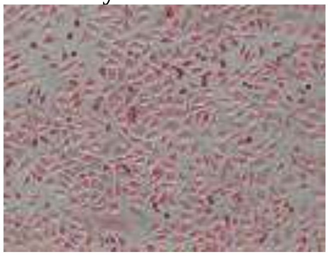

T_3 - sampled after 60 days
